# Electrochemical Lensing for High Resolution Nanostructure Synthesis in Liquids

**DOI:** 10.1021/acsanm.4c02295

**Published:** 2024-06-24

**Authors:** Auwais Ahmed, Peter A. Kottke, Andrei G. Fedorov

**Affiliations:** † George W. Woodruff School of Mechanical Engineering, Georgia Institute of Technology, Atlanta, Georgia 30332, United States; ‡ George W. Woodruff School of Mechanical Engineering and Parker H. Petit Institute for Bioengineering and Biosciences, Georgia Institute of Technology, Atlanta, Georgia 30332, United States

**Keywords:** electrochemical lensing, focused electron beam induced deposition, liquid phase nanostructure synthesis, silver nanomaterial, nanopillars

## Abstract

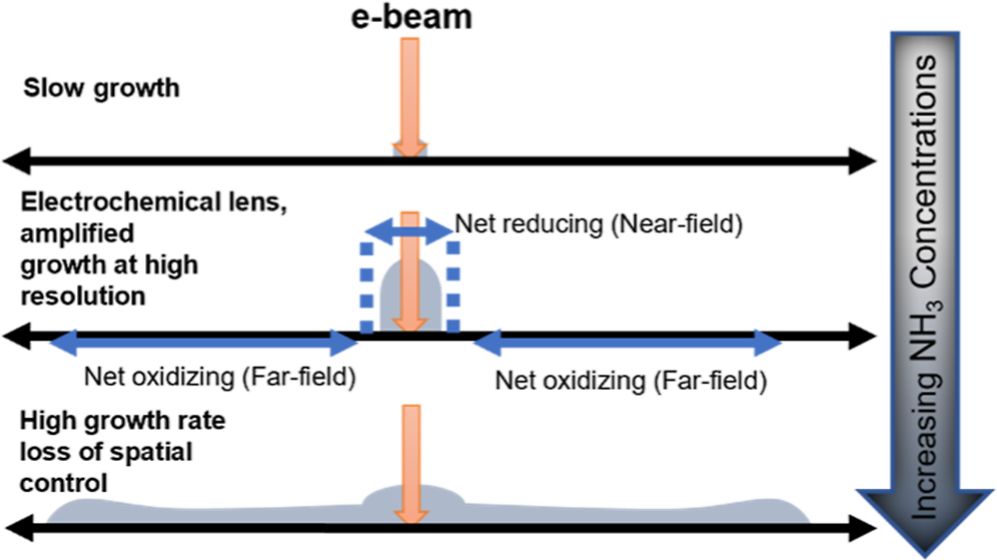

The advancement of liquid phase electron/ion beam induced deposition has enabled an effective direct-write approach for functional nanostructure synthesis with the possibility of three-dimensional control of morphology. For formation of a metallic solid phase, the process employs ambient temperature, beam-guided, electrochemical reduction of precursor cations, resulting in rapid formation of structures, but with challenges for retention of resolution achievable via slower electron beam approaches. The possibility of spatial control of redox pathways via the use of water–ammonia solvents has opened avenues for improved nanostructure resolution without sacrificing the growth rate. In particular, ammonia enables “electrochemical lensing” in which a tightly confined and highly reducing environment is created locally to enable high resolution, rapid beam-directed nanostructure growth. We demonstrate this unique approach to high resolution synthesis through a combination of analysis and experiment.

## Introduction

Techniques using focused electron beams generally have the potential to lead to the highest-resolution nanostructure synthesis. Extensive research has been conducted on the application of the focused electron beam for the fabrication of nanostructures, demonstrating its effectiveness, precision, and versatility across nano- and microscales.^1^ The utility of focused electron beam induced deposition (FEBID) has been shown in material science applications, including nanoelectronics, optics, nanoprobes, plasmonics, and magnetics.^2−4^ Beam-based fabrication provides the ability to create nanostructures atom-by-atom on suspended atomically thin substrates with minimal parasitic codeposition effects.^1^ However, this potential for “spot-on” synthesis is yet to be fully realized, especially in an environment that meets requirements relevant to practical applications. The most commonly used FEBID approach is gas phase electron beam induced deposition. The process has been employed to create metallic deposits, including silver,^5,6^ gold,^7^ copper^8^ and ruthenium,^9^ and has been combined with other deposition techniques to create 3D complex structures.^10,11^ Gas phase FEBID, however, suffers from low growth rate and impurities due to parasitic carbon entrapment.^4^ Recent reviews provide a comprehensive overview of the advancements and challenges associated with gas phase FEBID, highlighting various strategies to mitigate these issues and improve the quality and efficiency of the deposition process.^12^ Increasing growth rates often leads to unwanted deposition, compromising resolution,^13^ as, in direct-write nanofabrication, there is a general trade-off between growth rate and resolution. In this work, we describe an approach to resolve this fundamental trade-off between growth-rate and resolution, with a technique we call “electrochemical lensing”. Electrochemical lensing exploits the principles of electrochemistry and transport to facilitate high growth rates without sacrificing resolution.

Use of liquid phase precursors for FEBID (LP-FEBID) has been shown to offer higher growth rates, higher purity, and a greater selection of precursors as compared to gas phase precursors.^14^ For high resolution FEBID, the process takes place inside a vacuum chamber, where rapid evaporation makes the use of liquid phase precursors challenging. Enclosed cells, with electron transparent membranes, can be employed to isolate the liquid from the vacuum environment and are useful for fundamental studies.^15−18^ However, for practical applications that demand precise control of precursor concentration, diversity of substrate selection, and the creation of complex three-dimensional nanostructures, electron beam deposition in a free-surface liquid film offers many advantages and flexibility despite the challenges inherent in creating a film in a vacuum. In one free-surface approach, LP-FEBID has been applied to the bulk liquid layer formed via condensation in an environmental (low vacuum) scanning electron microscope (ESEM).^19,20^ Combinations of ESEM with microwells^19^ and liquid injected via nanocapillaries^21^ have also been used to create thick liquid films for FEBID. In these approaches, the thickness of the liquid film has proven problematic, as the beam electrons cannot penetrate the liquid and deposits are strongly affected by the moving liquid, often failing to even adhere to the substrate. The development of NanoElectroSpray-Assisted Focused Electron Beam Induced Deposition (NESA-FEBID), on the other hand, addresses this difficulty as it enables creation of a stable and ultrathin liquid film in high vacuum.^22,23^ Recent studies using NESA-FEBID have demonstrated that the choice of solvent for LP-FEBID can have significant effects on the chemistry of the process, with implications for both ease and rapidity of nanostructure synthesis. For instance, the use of a pure water solvent has been shown to be challenging for creating the stable liquid films necessary for spatial control of beam-guided synthesis and can also lead to a highly oxidizing electrochemical environment, slowing growth and thus minimizing the inherent advantage of LP-FEBID.

The addition of ammonia to an aqueous solution has been shown to promote nanomaterial synthesis due to its ability to make the chemical environment more reducing.^24^ The presence of ammonia in water leads to an environment around the primary e-beam irradiation site that is highly reducing for metal cations (favorable for electrochemically induced metal deposition) due to (a) the switching of the behavior of the radiolytically generated hydrogen peroxide from purely oxidizing to dual reducing/oxidizing, and (b) locally depressed concentrations of short-lived oxidizing species from radiolysis consumed by NH_3_ and NH_2_^•^ that act as scavengers of oxidizing species.^24^ However, high ammonia concentrations can also lead to fast growth of unwanted codeposits away from the desired nanostructure formation location. Controlling the spatial distribution of the solid matter deposited in and around the primary electron beam irradiation site is mediated by species transport and is crucial for achieving high resolution in electron beam nanostructure synthesis for direct-write applications.

A promising strategy in spatial control of electron beam guided synthesis is to take advantage of a water–ammonia solvent that creates a reducing radiolysis environment in the immediate vicinity of primary electron beam impingement, i.e., the location of a high electron dose rate (the “near-field” region). At the same time, an oxidizing environment (such as that present when pure water is the solvent) could be established outside of the primary beam impingement site or the “far-field” region. Exploiting the interplay between the redox species transport and reactions in this dual-domain environment enables an “electrochemical lensing” effect, where the growth of nanostructures is precisely “focused” and amplified at the near-field, while unwanted growth in the far-field is suppressed.

Uncovering and quantifying the interplay between the reaction and transport of chemical species that yield the electrochemical lensing phenomenon is the focus of this paper, which is organized as follows. First, the NESA-FEBID synthesis is described, focusing on the physicochemical state of the unconfined liquid film exposed to vacuum. Then we describe relevant chemical pathways taking place in the ammonia–water solution with the dissolved silver nitrate precursor during electron beam irradiation. A reaction-transport model is presented, and insights from simulations are used to reveal the mechanisms resulting in the desired electrochemical lensing effect. Finally, the results of simulation are validated using corroborating experiments and generalized to broader conclusions relevant to other electrochemical synthesis methods.

## Results and Discussion

### FEBID Synthesis in NESA Films

We use a NESA-FEBID process with ammonia–water as the solvent and dissolved silver nitrate (AgNO_3_) salt as a source of cations for Ag deposition. The presence of ammonia in the solution leads to a rapid equilibrium between Ag^+^ ions and silver diamine ion complexes, Ag(NH_3_)_2_^+^, eq 1, which favors greater Ag(NH_3_)_2_^+^ concentrations with increasing ammonia content.
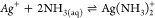
1

**Figure 1:**
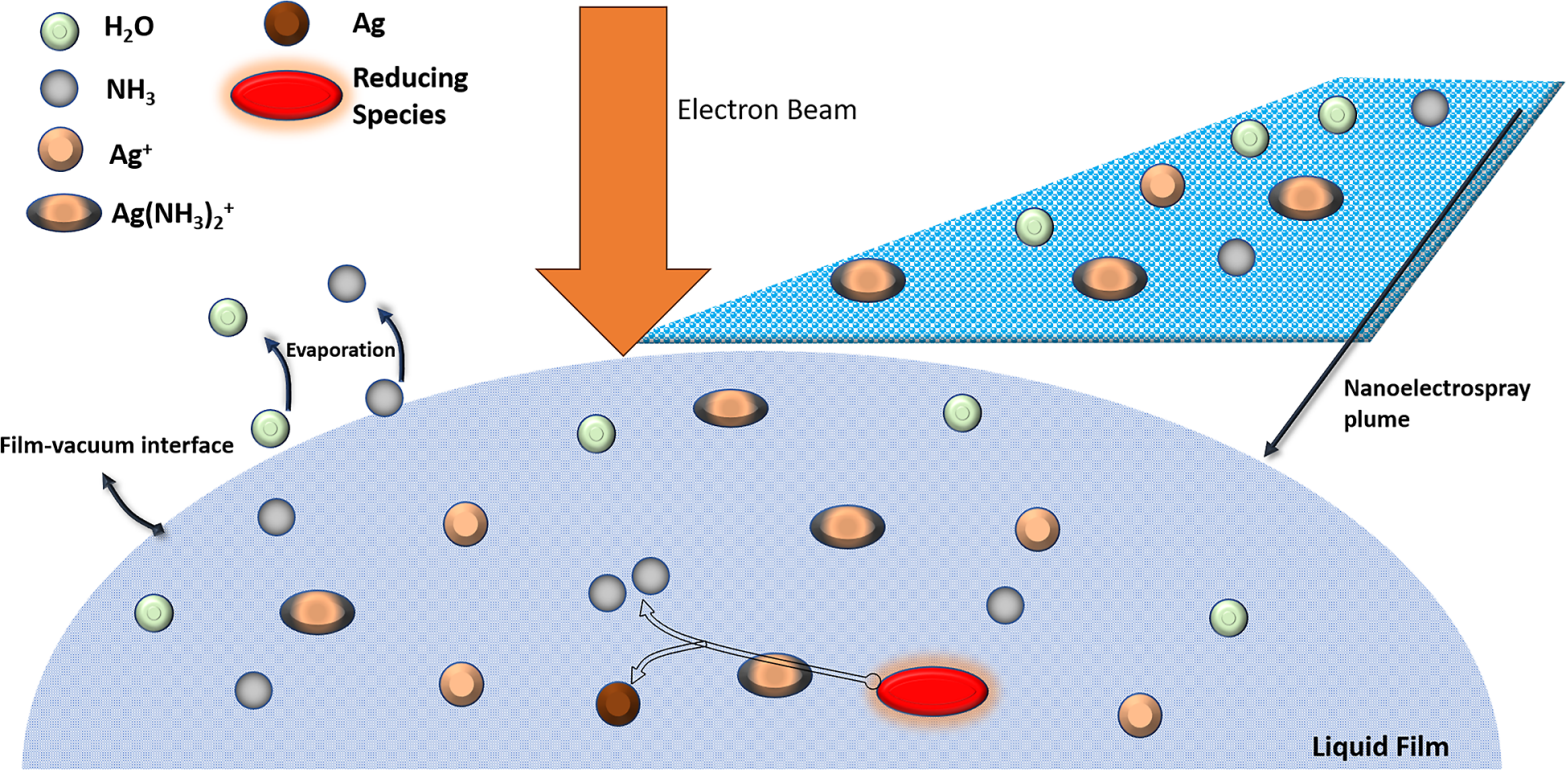
Solution of water and ammonia as solvent containing Ag^+^ and the Ag(NH_3_)_2_^+^ precursor is nanoelectrosprayed onto the substrate and forms a film. As the solution is supplied to the substrate, the film size increases. Water and ammonia evaporate from the film surface slowing the growth of the film. The film reaches a quasi-steady state size and ammonia concentration as the evaporation balances the addition of solution via nanoelectrospray. The concentrations of nonvolatile Ag^+^ and Ag(NH_3_)_2_^+^ precursors increase continuously (i.e., do not achieve a steady state value). The presence of Ag(NH_3_)_2_^+^ provides an additional source of stored ammonia, as a single Ag(NH_3_)_2_^+^ ion-complex releases two ammonia molecules if it is reduced to Ag.

In NESA-FEBID, Figure 1, irradiation of the liquid film by the electron beam results in the radiolytic production of chemically reactive species. High energy electrons undergo elastic and inelastic collisions with the solvent molecules. This results in a transfer of energy from the electron beam to the liquid and leads to radiolysis, resulting in the creation of primary radiolytic species such as solvated electrons, e_sol_^–^, as well as H^•^, H_2_O_2_, H_3_O^+^, OH^•^, HO_2_^•^, NH_2_^•^, and H_2_.^25−27^ These primary radiolytic species then undergo reactions with each other and the solution, leading to the creation of secondary radiolytic species such as O_2_ and N_2_H_4_.^24,28^ Both primary and secondary radiolytic species diffuse away from their region of formation to react with the precursor, the solvent, and one another in both the near-field and far-field regions of the film.

### Reaction Pathways Essential to Nanomaterial Synthesis

**Figure 2:**
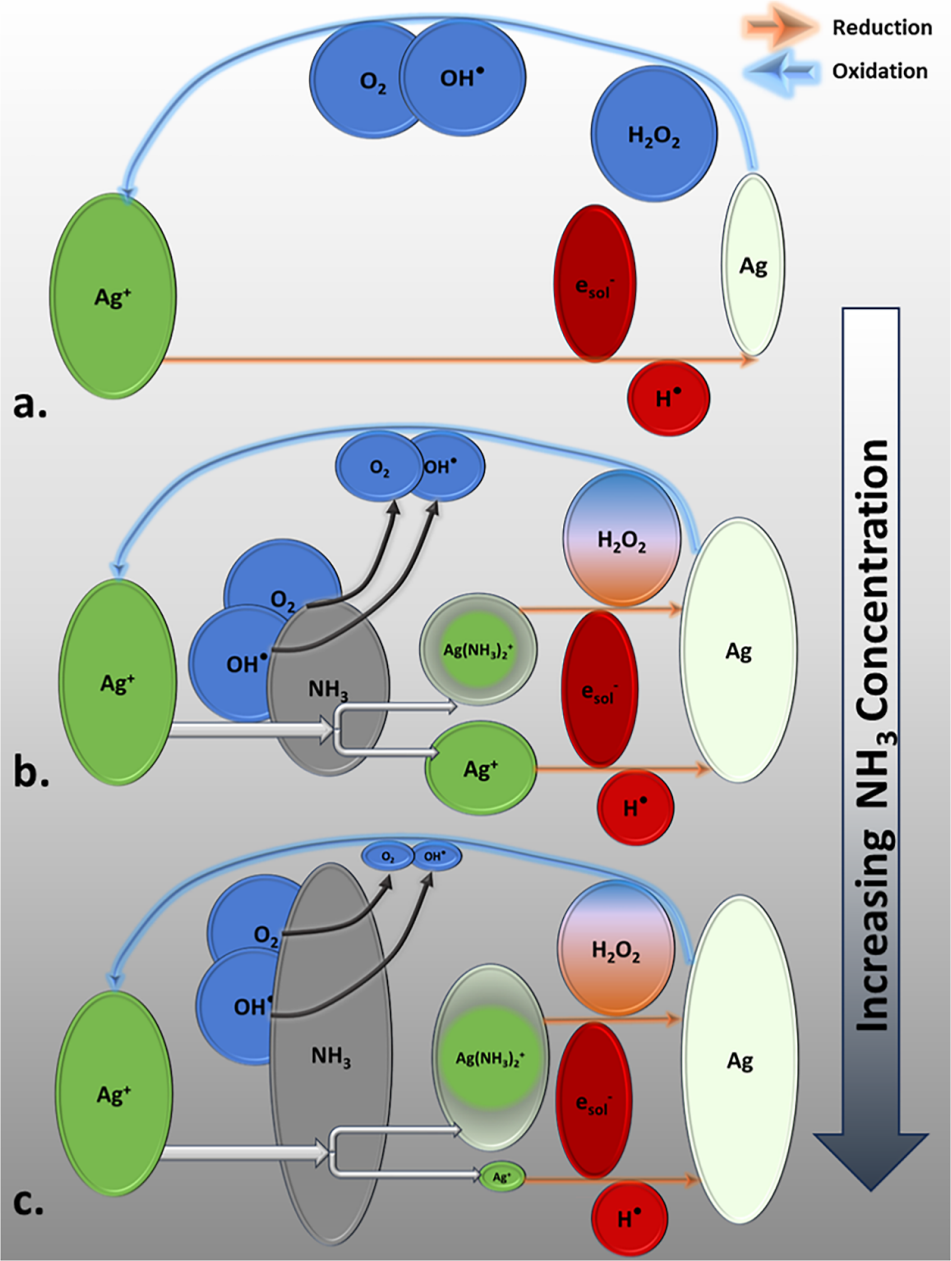
Important chemical pathways for silver production in LP-FEBID with an ammonia–water solvent and metal salt precursors (shown in green). The reducing species are illustrated in red, and oxidizing species in blue. In the absence of NH_3_ (a), the precursor Ag^+^ is converted to Ag via reduction by solvated electrons. The Ag can then be reverted to Ag^+^ via oxidation. The addition of ammonia to solution (b) leads to partial conversion of Ag^+^ to Ag(NH_3_)_2_^+^. This adds an additional silver formation pathway, as Ag(NH_3_)_2_^+^ can be reduced to Ag by H_2_O_2_. The role of H_2_O_2_ is shifted from purely an oxidizer of Ag to both an oxidizer and a reducer. Presence of NH_3_ also depletes the concentrations of oxidizing species O_2_ and OH^•^, making the environment more reducing. Further addition of NH_3_ (c) amplifies this effect, leading to the environment becoming even more reducing. The size of each species oval indicates their relative concentrations. At intermediate ammonia concentrations (b) yields the relative amounts of the two precursor species and the oxidizers that provide the optimum conditions for high growth/high resolution deposition, i.e., electrochemical lensing.

In the NESA-FEBID of AgNO_3_/ammonia–water, silver metal formation occurs through the reactions shown in Figure 2, which highlights the impact of changing ammonia concentration. In an environment in which pure water serves as the solvent (Figure 2a), an overall oxidative condition prevails, which significantly inhibits the rapid growth of nanomaterials. The species of greatest significance for the synthesis of Ag nanomaterials are the ones that either reduce Ag^+^ and Ag(NH_3_)_2_^+^ to Ag or oxidize Ag to Ag^+^. In the absence of NH_3_, Ag^+^ from dissociated AgNO_3_ is reduced to Ag by solvated electrons e_sol_^–^ and hydrogen radicals H^•^. The Ag formed can then be converted back to Ag^+^ by oxidizing species, H_2_O_2_, OH^•^ and O_2_.^29^ The net rate of Ag creation is determined by the difference in rates of silver cation reduction and silver atom (solid phase) oxidation (Figure 2a). The introduction of ammonia changes the deposition landscape in multiple ways (Figure 2b).

First, in the presence of ammonia, the silver ions (Ag^+^) are partially converted to silver diamine, Ag(NH_3_)_2_^+^. This introduces an additional reduction pathway to solid Ag formation, where H_2_O_2,_ a primary radiolytic species, shifts from being exclusively an oxidizer for Ag to also reducing precursor Ag(NH_3_)_2_^+^ to form silver.^30^ Second, NH_3_ leads to the consumption of the oxidizing species OH^•^ and O_2_, leading to their diminished concentration.^31^ The net effect is a significantly enhanced rate of solid Ag deposit creation through both an increase in the reduction to Ag and a decrease in oxidation of Ag. Ag(NH_3_)_2_^+^ also acts as an additional source of ammonia as each ion-complex releases two ammonia molecules into the film upon reduction (Figure 1). Increasing the ammonia concentration further amplifies these effects (Figure 2c). In fact, at sufficiently high ammonia concentrations, nearly all dissolved silver precursor is in Ag(NH_3_)_2_^+^, enhancing the role of H_2_O_2_ as a reducer, and as the oxidizing species are highly suppressed, the resulting higher net growth rates of Ag eventually come with a loss of resolution. Reaction-diffusion simulations were used to obtain detailed insights into the processes leading to variation of net reducing versus oxidating behavior as a function of spatial location and time and thus identify the conditions in which high growth rate occurs without loss of deposit resolution. Supporting Information Section S2 describes the transient mass conservation and species transport model used to simulate the dynamic interplay between chemical reactions and the diffusion of species within the system.

During a NESA-FEBID process, sprayed nonvolatile species concentrations will continuously increase, while volatile species concentrations reach a steady state (Figure 1). We consider two initial silver salt concentrations, 26.5 and 265 mM, which are the concentrations occurring at 1 and 10 s, respectively, within a film of 50 μm radius and 1 μm thickness formed via introduction of a 250 μM AgNO_3_ solution at a rate of 3 μL/h. Initial free (not bound in silver diamine) dissolved ammonia concentrations in the film vary from ∼100 μM up to 3 M, which corresponds to the steady-state concentration achieved assuming evaporation at the kinetic limit to vacuum from a film of 50 μm radius and 1 μm thickness formed via introduction of a 5 to 30% w/w ammonia concentration in water at a rate of 3 μL/h (typical experimental conditions). The description of how the initial conditions for AgNO_3_ and NH_3_ concentrations are set is provided in Section S2 of the Supporting Information. The initial concentration of silver ions and silver diamine ions is based on the total initial AgNO_3_ concentration, the initial ammonia concentration, and the equilibrium condition, eq 1.

### Electrochemical Lensing: Insights from Simulations

**Figure 3:**
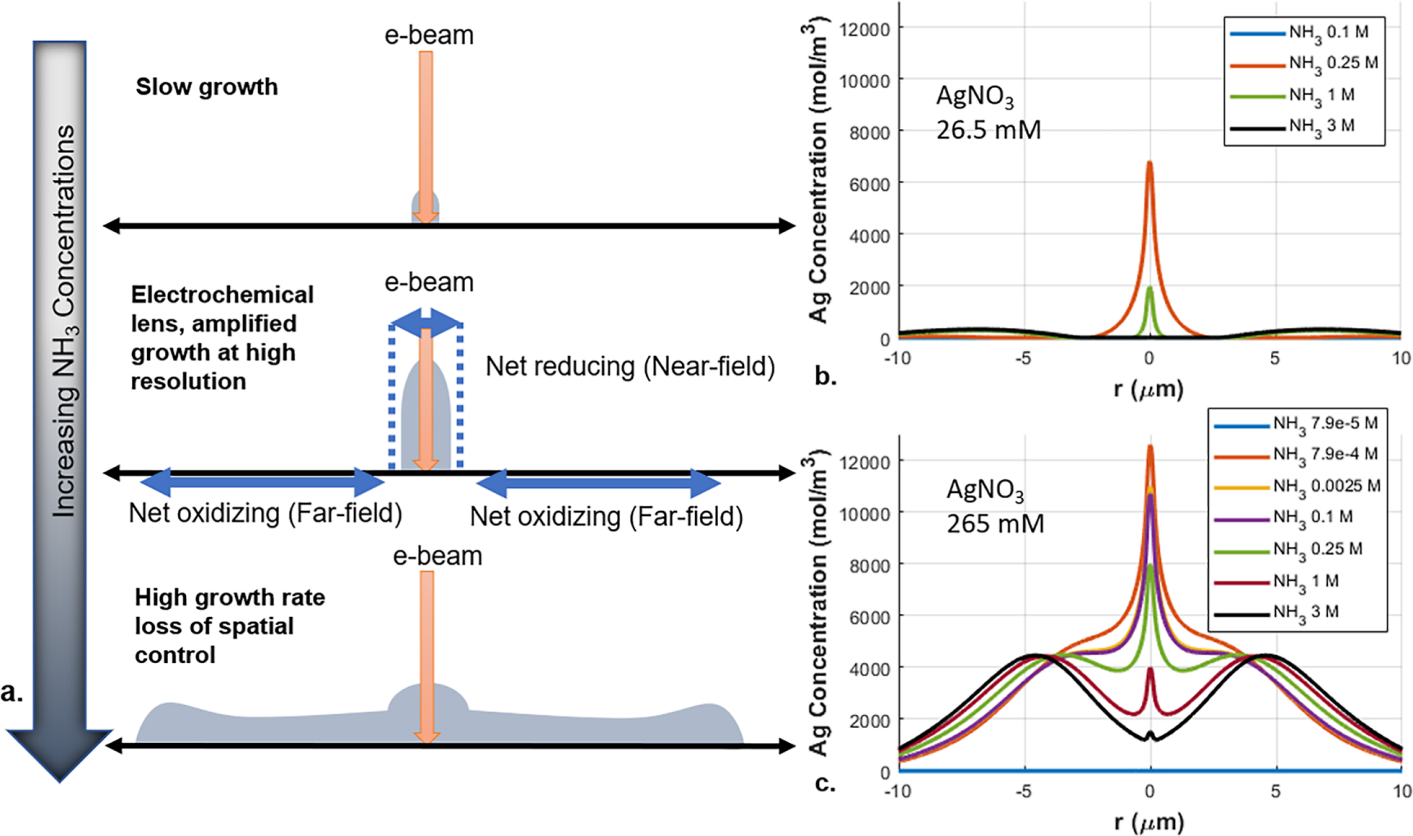
(a) Nanostructure deposition in water–ammonia solvents. In pure water or low ammonia, minimal nanostructure formation occurs due to oxidizing conditions. Optimal ammonia concentrations for electrochemical lensing result in focused and rapid nanostructure growth with high resolution, as reducing conditions prevail in the near-field and oxidizing conditions prevent nanostructure formation in the far-field. High ammonia concentrations lead to rapid growth of solid phase throughout the domain with loss of resolution. Panel (b) shows the simulated axisymmetric (mirrored around *y*-axis) Ag concentration profiles, after 1 s electron beam irradiation across different NH_3_-water solution concentrations Ag concentration profile in a 26.5 mM AgNO_3_ film, highlighting optimal NH_3_ concentration for electrochemical lensing at 0.25 M NH_3_ Panel (c) illustrates the 265 mM AgNO_3_ scenario, where increased precursor availability leads to substantial far-field deposition even at very small NH_3_ concentrations in the film, resulting in broadening growth and diminished resolution.

Electrochemical lensing is the phenomenon in which the transport and reaction interaction result in preferential growth in the near-field while minimizing nanostructure deposition in the far-field, a phenomenon which can be exploited to achieve high resolution deposits, as shown schematically in Figure 3a (middle panel).

To demonstrate the effect of the ammonia content on deposition, we perform simulations at multiple ammonia concentrations. The key result of simulations is the concentration of Ag, which serves as a proxy for silver nanostructure growth. Figure 3b,c shows the resulting Ag concentrations for various NH_3_ initial concentrations after a 1 s exposure of the film to a 30 kV electron beam.

For the lower silver precursor concentration, 26.5 mM, electrochemical lensing is observed at an intermediate ammonia concentration (Figure 3b). Negligible silver formation is observed after 1 s of beam exposure when the initial free NH_3_ concentration is 0.1 M: for this precursor concentration and small initial concentration of free ammonia, the entire domain remains net oxidizing for silver. When the NH_3_ concentration is increased to 0.25 M, the near-field environment becomes reducing, and Ag growth commences at the point of electron beam impingement, while the far-field continues to be oxidizing, thus preventing Ag production away from the e-beam irradiation. This distribution of Ag formation, resulting from creation of a reducing environment in the near-field only, is the hallmark feature of electrochemical lensing. As the NH_3_ concentration is further increased to 1 M, both the near-field and far-field environments become reducing, resulting in broadening of the area of net silver reduction. The strongly reducing environment not only leads to a higher rate of conversion of Ag^+^ and Ag(NH_3_)_2_^+^ precursor ions into Ag in the far-field but also, in turn, decreases the availability of these precursor ions for diffusion toward the near-field. Consequently, the central Ag pillar experiences a precursor shortage, slowing its growth compared to the rate observed at 0.25 M NH_3_. This effect is even more pronounced for higher concentrations of ammonia: for the initial NH_3_ of 3 M, significant far-field Ag formation occurs while central deposit formation is very limited.

Electrochemical lensing is not observed for all precursor silver salt concentrations. When the initial AgNO_3_ concentration is 265 mM, even very small initial concentrations of free ammonia correspond to rapid and widespread Ag formation (Figure 3c). In part, this is due to the presence of significant “bound” ammonia locked in the Ag(NH_3_)_2_^+^ complex. When silver diamine is reduced, NH_3_ enters the solution, resulting in an increase of ammonia concentrations (Figure 1). Thus, while at extremely low initial free ammonia levels (79 μM), silver growth is negligible, high rates of near-field deposition are achieved even with only 0.79 mM of initial free ammonia. However, the lensing effect that occurs at the lower salt concentration is diminished, with significant net reduction occurring outside the near-field region, as NH_3_ released during Ag(NH_3_)^2+^ reduction diffuses to the far-field. At higher ammonia concentrations (2.5 mM to 3 M), silver growth is even more pronounced in the far-field, leading to formation of volcano-like structures (Figure 3c).

**Figure 4:**
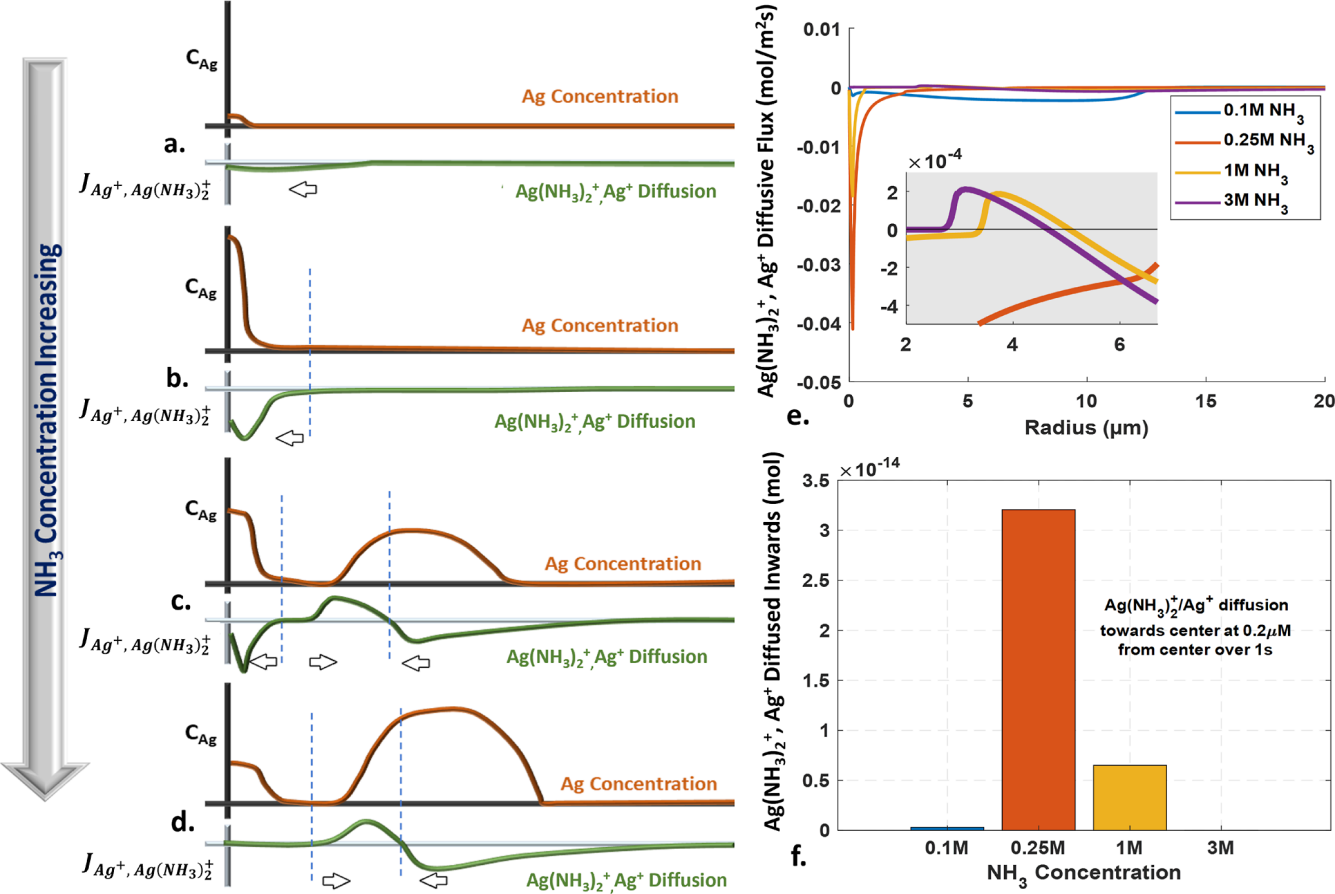
Impact of NH_3_ concentration on Ag^+^/Ag(NH_3_)_2_^+^ precursor diffusion and Ag creation. (a–d) Schematic illustration of the effect of increasing ammonia concentrations on diffusion of precursor in the domain and resulting Ag creation. In low NH_3_ or pure aqueous conditions (a), oxidizing conditions inhibit Ag formation with minimal precursor diffusion. At optimal NH_3_ concentration (b), a reducing environment in the near-field promotes precursor cations Ag^+^ and Ag(NH_3_)_2_^+^ conversion to Ag, while maintaining oxidizing conditions in the far-field, promoting precursor cation diffusion toward the near-field. Higher NH_3_ concentrations (c,d) lead to Ag^+^ and Ag(NH_3_)_2_^+^ conversion in both the near-field and far-field, decreasing precursor cations available for diffusion toward near-field and slowing Ag growth. Panel (e) shows the simulated diffusive flux profiles for the precursor over the domain for various NH_3_ concentrations under a 30 kV electron beam for 1 s. Panel (f) depicts the total diffusion of Ag^+^ and Ag(NH_3_)_2_^+^ toward the near-field at a distance of 0.2 μm from the center over a time of 1 s.

Section S3 of the Supporting Information reveals the details of the fundamental principles for the observed deposition behavior as a function of ammonia and silver salt concentration. Figures S2 and S3 depict key reaction rates and concentrations in the near-field (center) and farfield (4.5 μm from the center), respectively, 1 s after electron beam irradiation of a 26.5 mM silver nitrate film for four representative initial ammonia concentrations. Figure 4 depicts in greater detail the role of transport in silver deposition rates at different locations. A film consisting of extremely low (or no) NH_3_, Figure 4a, will lead to an overall oxidizing environment. This yields very little or no creation of Ag anywhere. Additionally, very little conversion of Ag^+^ and Ag(NH_3_)_2_^+^ to Ag causes a very small concentration gradient of Ag^+^ and Ag(NH_3_)_2_^+^ and consequently a very small diffusion flux. At the optimum NH_3_ concentration, Figure 4b, reducing conditions are established at the near-field, while conditions at the far-field remain oxidizing. This leads to conversion of precursor, primarily Ag(NH_3_)_2_^+^, to Ag in the near-field, with the precursor supplied to the near-field by diffusion from the far-field. At higher NH_3_ concentrations, Figure 4c,d, the precursor is reduced to Ag both in the near-field and far-field. This leads to growth of Ag in the far-field, as well as slowing down of growth of Ag in near-field by decreasing the diffusion of precursor toward the near-field. The ammonia concentration (0.25 M) associated with the highest magnitude diffusion flux is the one at which electrochemical lensing effect occurs. Figure 4e shows the simulated diffusive flux profiles of the Ag^+^/Ag(NH_3_)_2_^+^ precursor in the domain at 1 s. The highest diffusive flux toward the nearfield is observed at 0.25 M NH_3._ Increasing ammonia to 1 M decreases the precursor flux toward the center, resulting in a slower central pillar growth. Further increase to 3 M NH_3_ amplifies this effect. Figure 4f shows the total amount of the precursor delivered toward the center over 1 s from a distance of 0.2 μm (a proxy for the deposit size). The greatest precursor diffusion occurs at 0.25 M ammonia concentration, resulting in electrochemical lensing with amplified Ag deposition in the nearfield.

### Electrochemical Lensing—Corroborating Experiments

**Figure 5:**
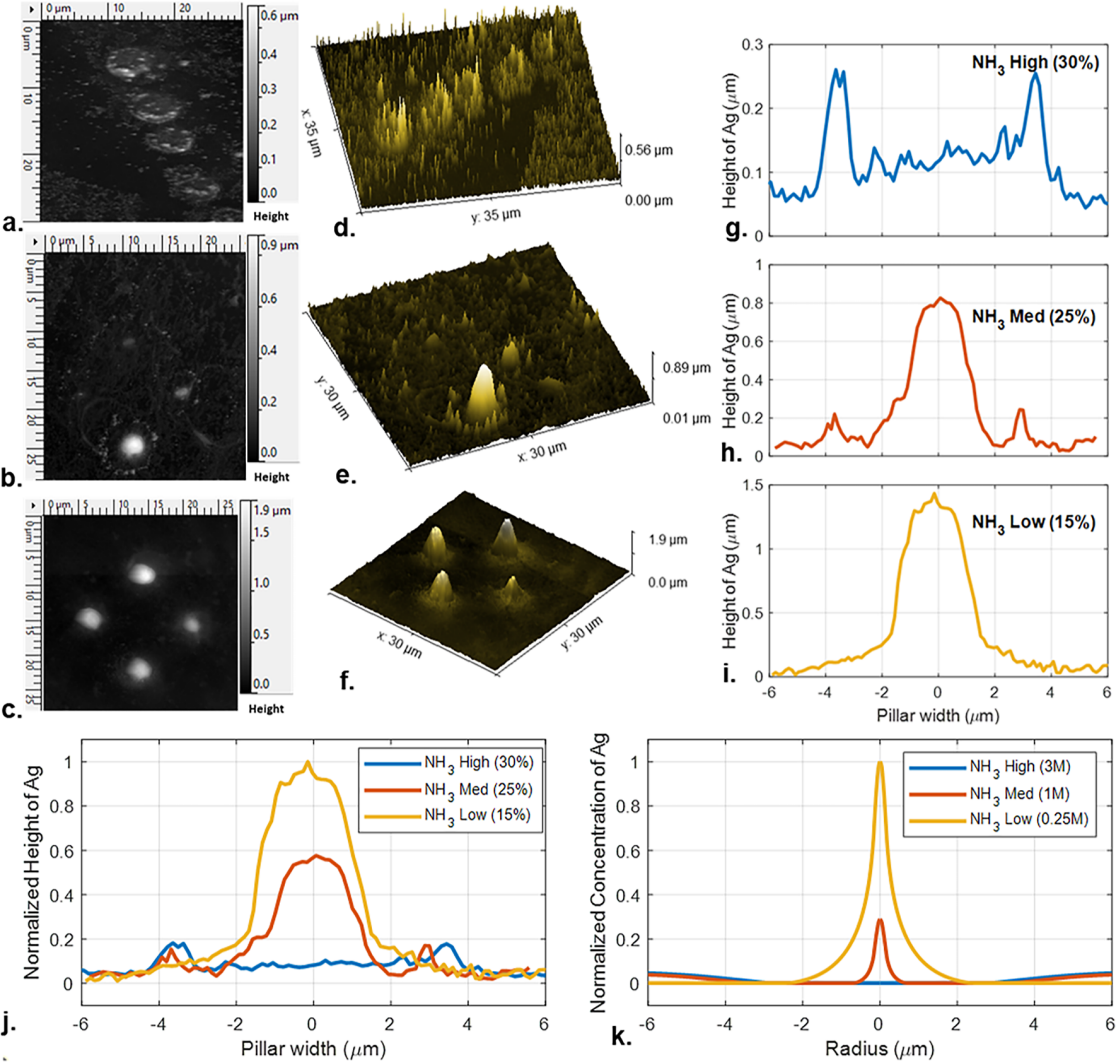
AFM images of nanostructures from NESA-FEBID using water–ammonia solutions with 250 μM AgNO_3_ at 30, 25, and 15% NH_3_ concentrations in top-view 2D (a–c) and 3D (d–f) radial profiles (g–i). Nanostructures are created with 1s 30 kV electron beam in “spot” mode. Ag growth varies with NH_3_ concentration, showing focused near-field growth at 15% NH_3_ and increased far-field growth at 25 and 30% NH_3_. Normalized experimentally determined pillar profiles (j) are compared to simulated Ag concentration profiles at varying NH_3_ levels (k), showing qualitative agreement and the presence of optimal NH_3_ concentrations for focused near-field Ag growth, the signature of the electrochemical lensing.

To corroborate our simulation results, we conducted a series of experiments using NESA-FEBID with a 250 μM AgNO_3_ solution, under a 30 kV electron beam for 1 s. We explored the effects of varying NH_3_ concentrations in the solvent. Attempts to form silver deposits using AgNO_3_ dissolved in pure water (no ammonia) were not successful, supporting the simulation predictions of the suppression of Ag growth in a highly oxidizing environment. Line and square arrays of nanostructures (to ensure repeatability) deposited from initial 30, 25, and 15% ammonia–water NESA-FEBID solutions (corresponding to 3, 1, and 0.25 M ammonia concentrations in the film) were analyzed via atomic force microscopy (AFM). Figure 5a–i show both two- and three-dimensional images and the radial profiles of these nanostructures. To compare the experimental results with the simulations, normalized pillar heights from the experiments, Figure 5j, were compared to the normalized Ag concentration profiles obtained for different film NH_3_ concentrations from the simulations, Figure 5k.

Experimental silver creation follows the expected behavior with negligible silver growth in pure water and a silver peak created in the near-field at 15% NH_3_ NESA-FEBID solution with very little far-field growth. Increasing the concentration of NH_3_ to 25 and 30% in the NESA-FEBID solution led to higher Ag growth in the far-field and slower Ag growth in the near-field. This trend, predicted by simulations and confirmed by experiments, demonstrates the electrochemical lensing concept obtained when a metal precursor cation is converted into its diamine complex, enabling radiolytically produced H_2_O_2_ to act as a dual reducer and oxidizer. This leads to a high growth rate synthesis of high aspect ratio nanostructures in the near-field with corresponding increase in resolution. The experimental methods are detailed in Section S2 of the Supporting Information.

## Conclusions

We present an approach to resolving the fundamental trade-off between the growth rate and resolution in electron beam mediated direct-write nanofabrication through the technique of electrochemical lensing. When deposition processes are governed by systems with a linear behavior, the increase in the concentration of a reactant, even locally at the desired deposition location, will result in a broader deposition region due to diffusion. Electrochemical lensing is accomplished via introduction of intentional nonlinearity into the system. Metal nanostructure synthesis using FEBID from silver salt in a water–ammonia solution is a demonstration of electrochemical lensing, in which the strong nonlinearity of the system’s response to changing ammonia concentration comes from the multiple roles played by ammonia in the system: (a) by binding silver cations into diamine silver complexes, ammonia adds a new reduction pathway involving hydrogen peroxide and provides a mechanism for ammonia storage in the solution; and (b) by using both ammonia and its radiolytic decomposition product NH_2_^•^ to selectively scavenge oxidizing species. Generalization of the approach therefore requires seeking similarly complementary nonlinearities for exploitation through use of a solvent for forming multiple forms of a cation whose reduction leads to solid formation or encoding the redox switchable behavior to one or more transient intermediary species that facilitates the synthesis reaction. Electrochemical lensing as a conceptual model for synthesis improvement could play a significant role in a wide variety of nanomanufacturing applications, offering a scalable, precise, and efficient pathway for the creation of nanostructures with greater control of the shape and resolution.

## Supporting Information Available

The Supporting Information document consists of experimental methods, details of the reaction and transport model, and additional simulation results and their explanation to further understand electrochemical lensing phenomenon (PDF)
