# Supplementary material for: Electrochemical Lensing for High Resolution Nanostructure Synthesis in Liquids
Source: ACS Appl Nano Mater. 2024 Jun 24;7(13):15438–45. doi: 10.1021/acsanm.4c02295 (PMC11249771; doi:10.1021/acsanm.4c02295)
Supplement: Supplementary file 1 — an4c02295_si_001.pdf [file an4c02295_si_001.pdf]

Supporting Information

Electrochemical Lensing for High Resolution Nanostructure Synthesis in Liquids

Auwais Ahmed,[a] Peter A. Kottke,[a] Andrei G. Fedorov [a,b]*

[a] George W. Woodruff School of Mechanical Engineering, Georgia Institute of Technology, 771 Ferst Dr NW, Atlanta, GA 30332, USA

[b] Parker H. Petit Institute for Bioengineering and Biosciences, Georgia Institute of Technology, 771 Ferst Dr NW, Atlanta, GA 30332, USA

* Corresponding Author: Andrei G. Fedorov, Email: AGF@gatech.edu

Contents of Supporting Information:

Sections

S1 Experimental Methods

S2 Reaction and Transport Model

S3 Electrochemical Lensing: Insights from Simulations

Figures S1-S3

S1: Experimental Methods

HPLC grade deionized water (Sigma Aldrich) and an HPLC grade water-ammonia solution (Fisher) were used for all experiments. Silver nitrate salt (Sigma Aldrich) was dissolved in water and mixed with water-ammonia to achieve desired concentrations. A TA Hamilton 1750 TLL syringe was used to deliver the solution at 3 μL/hr via a syringe pump. The solution nanoelectrospray was carried out using a fused silica capillary emitter (360 μm outer diameter, 100 μm inner diameter, pulled to a 3±1 μm diameter tip using a Sutter P-2000 Laser-Based Micropipette Puller). The substrate was a 1 cm x 1 cm square Si wafer coupon with a 100 nm gold coating. The capillary to substrate distance is maintained at ~200 μm. The negative mode nanoelectrospray was initiated by electrically biasing the solution with -500 V at the metallic union using a SRS 5 kV power supply. Experiments were conducted using the FEI Quanta 200 SEM operated in high vacuum mode (~10^{-4} torr). To distinguish nanostructure deposits from salt precipitates, Nanometer Pattern Generation System (NPGS) exposed the film in a predefined pattern of four 1-second spots, facilitating the formation of discernible nanostructures. Post-deposition, the samples were immersed in water for 2 hours and subsequently dried. The Bruker Dimension Icon was used to perform AFM imaging. Monte Carlo simulations were performed using the CASINO software.

S2: Reaction and Transport Model

A transient mass conservation and species transport model is used to simulate the dynamic interplay between chemical reactions and the diffusion of species within the system. The liquid film, depicted at the top of the diagram in Figure S1a, is very thin owing to the nanoelectrospray delivery, such that the rate variations across it could be ignored for a quasi-1D axisymmetric treatment (Figure S1b). Rather than introduce assumptions regarding the solid phase nucleation

process, we use the Ag concentration as a proxy for nanostructure creation.[24] This allows us to treat solid metal reduction and oxidation as homogeneous processes with a correction factor accounting for heterogeneous (confined to the interface) nature of oxidation. Additionally, the

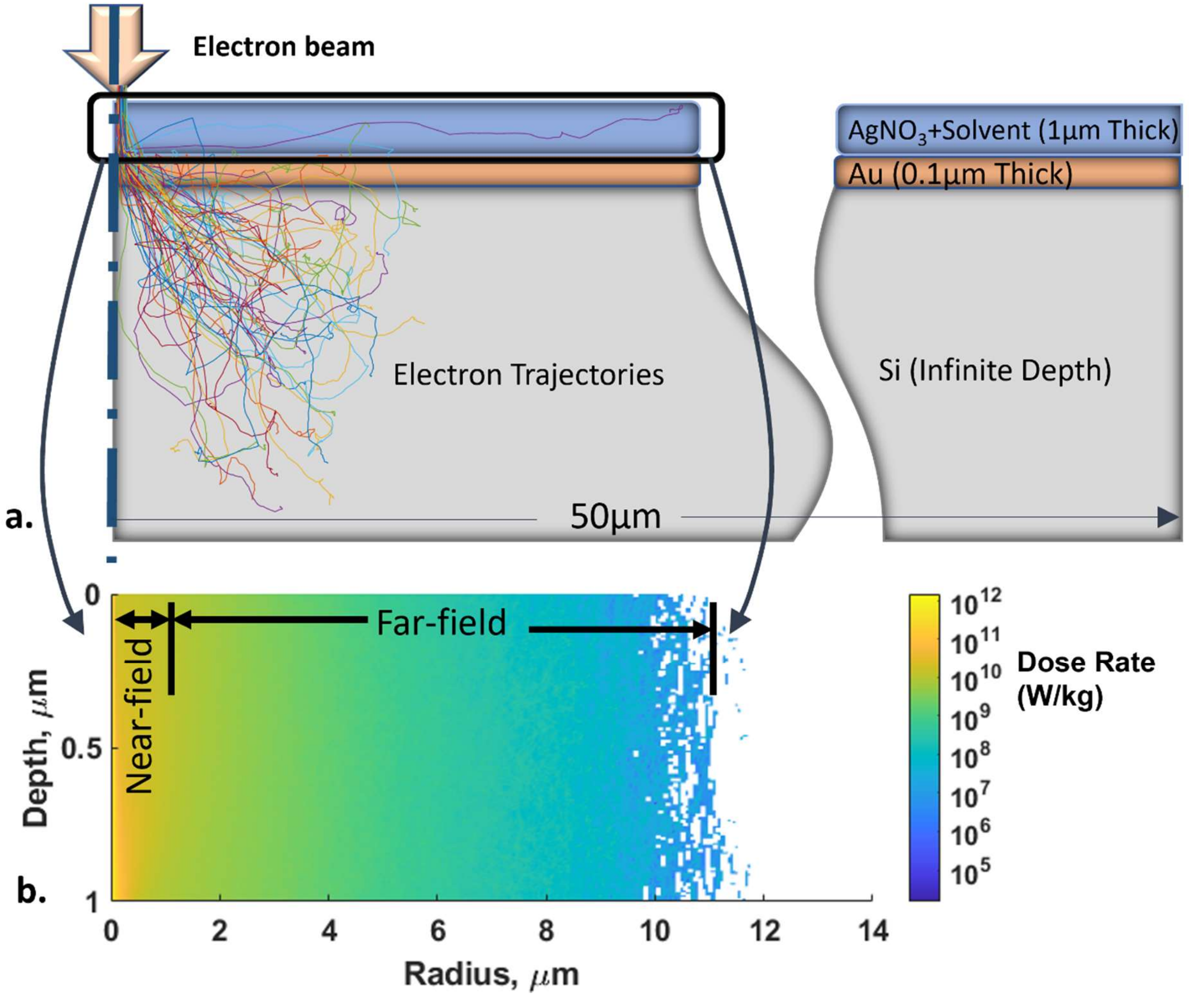

Figure S1. *Monte-Carlo simulation is used to calculate the dose rate distribution due to a 30 kV, 3 nA electron beam irradiation. (a) The simulation domain - a 1 μm thick liquid film is overlayed on a 0.1 μm gold layer, which is placed on a thick (treated as semi-infinite) silicon substrate. (b) The axisymmetric dose rate distribution within the liquid film. The origin (0,0) is the point of electron beam irradiation, and the axis of symmetry is the vertical line passing through this point. Due to negligible axial variation in dose rate, the problem can be simplified to 1D axisymmetric.*

effects of advection and electromigration are not considered, and Fickian diffusion is assumed. These assumptions have been shown to accurately capture the key processes and predict electron mediated metallic nanomaterial synthesis.[24]

The variation of the concentration of species i, C_i, with time and radial position, t and r, respectively, is given by,

$$\frac{\partial C_i}{\partial t} = D_i \frac{1}{r}\frac{\partial}{\partial r}(rC_i) + S_i + R_i \tag{S1}$$

D_i is the diffusion coefficient of species i, S_i is the net rate of production/consumption by homogeneous chemical reaction, and R_i is the net rate of production by radiolytic processes. The homogenous reactions are treated as elementary reactions, i.e.,

$$S_i = \sum\nolimits_p k_p \prod\nolimits_{j\in r_p} C_j^{s_j} - \sum\nolimits_c k_c \prod\nolimits_{m\in r_c} C_m^{s_m} \tag{S2}$$

where, k_p and k_c and the rate constants for the chemical reactions producing species i, r_p, and consuming species i, r_c, respectively.

The exponents s_j and s_m represent the associated stoichiometric coefficients. The source term representing net creation or consumption by radiolysis, R_i, is given by $R_i = G_i\psi$ where the G-value, G_i, is solvent dependent and gives the moles of species i created per unit energy of electron beam absorbed, and ψ is the local rate of energy deposition from e-beam irradiation, found using azimuthally and axially integrated values from Monte Carlo simulations implemented in CASINO, divided by thickness and circumference to obtain local average values for the 1-D simulations. The chemical reactions, the rate constants and G-values utilized are specified in our previous work.[24]

The reaction source term, S_i, as given in Eq.(S2), is for homogenous reactions; however, the metallic silver produced by the NESA-FEBID process will eventually nucleate to form a nanostructure, a process that cannot be modeled explicitly in the 1-D formulations. In growing nanostructures, only the Ag present on the solid deposit surface remains exposed and susceptible

for oxidation, via heterogeneous reactions. As a solid deposit increases in size its surface-to-volume ratio decreases, as does the proportion of Ag available for oxidation relative to the total amount of Ag locally present. To account for decreased availability of Ag for oxidation after nucleation, while maintaining the simplicity of a homogenous reaction model, we assume that the reaction rate of silver atoms on the surface of deposits follows the same rate expression as in the bulk, but only consider surface atoms as contributing to the concentration of Ag participating in the oxidation reaction. This approach is accomplished by modifying the form of the Ag oxidation expression with a dimensionless prefactor, *f*, that is a function of silver concentration: $f = \min(C^*/C_{Ag}, 1)$, where the value of the reference concentration C^* is based on the molar density of solid silver and the film thickness (1 μm), $C^* = 38.88\ \mathrm{mol/m^3}$. Zero flux in far field and axial symmetry boundary conditions are used on the two domain boundaries.

We consider two initial silver salt concentrations, 26.5 mM and 265 mM, which are the concentrations occurring at 1 s and 10 s. The concentration was calculated as follows,

$$C_f = \frac{1}{V_f}\int_0^t Q_{es}\, C_{es}\, dt \qquad \text{(S3)}$$

where C_f is the concentration of $AgNO_3$ in the film, Q_{es} is the flow rate for electrospray, C_{es} is the concentration of $AgNO_3$ in electrosprayed solution and V_f is the volume of the film. For the case under consideration a 250 μM solution of $AgNO_3$ was electrosprayed at a flow rate of 3 μL/hr to create a 50 μm radius and 1 μm height film. Using t=1 s as an upper limit for the integral, the concentration of $AgNO_3$ in the film is equal to 26.5 mM. For t=10 s, the concentration C_f=265 mM.

Due to the dynamic and non-linear nature of the process under vacuum conditions, it is very difficult to calculate the corresponding ammonia concentration in the film for a particular input nanoelectrosprayed ammonia concentration. A range of ammonia concentrations in the film was estimated using the kinetic limit to vacuum from a film of 50 μm radius and 1 μm thickness formed via the introduction of a 5% to 30% w/w ammonia concentration in water at a rate of 3 μL/hr (typical experimental conditions).

$$\frac{dN_{evap}}{dt} = \alpha \frac{p_{vap}}{\sqrt{\pi MRT}} A_{surf} \tag{S4}$$

$$\frac{dN_{es}}{dt} = Q_{es}\, C_{es} \tag{S5}$$

$$\frac{dN_{H_2O}}{dt} = \frac{dN_{es,H_2O}}{dt} - \frac{dN_{evap,H_2O}}{dt} \tag{S6}$$

$$\frac{dN_{NH_3}}{dt} = \frac{dN_{es,NH_3}}{dt} - \frac{dN_{evap,NH_3}}{dt} \tag{S7}$$

the molar flow rates into the film are represented as N_{NH_3}, N_{H_2O} and N_{es} for ammonia, water and electrospray, respectively. The molar rate of evaporation of water and ammonia from the film, N_{evap}, is given by Hertz-Knudsen equation Eq.(S4). The upper and lower limits of the ammonia concentrations in the film were estimated to lie between 0.1 M and 3 M.

The model was solved using COMSOL Multiphysics software.

S3: Electrochemical Lensing: Insights from Simulations

Simulations reveal details of the fundamental principles for the observed deposition behavior as a function of ammonia and silver salt concentration. Figure S2 depicts key reaction rates and concentrations in the near-field (center) 1 sec after electron beam irradiation of a 26.5 mM silver nitrate film for four representative initial ammonia concentrations. In addition to the instantaneous rate of silver creation (Figure S2a), which mirrors the results showing net silver produced in the near-field from Figure 3b, Figure S2b depicts the dominant reduction and oxidation reaction rates. Even at the lowest ammonia concentration, 0.1 M, solvated electron reduction of Ag^+ has been supplanted as the dominant Ag production route by H_2O_2 reduction of $Ag(NH_3)_2^+$.

The higher rate of silver production at 0.25 NH_3 ammonia is not due to higher rates of reduction but a more significant decrease in the rate of oxidation than in the rate of reduction. In particular, the consumption of oxidizing species $OH^\bullet$ and O_2 by NH_3 and radiolytically produced $NH_2^\bullet$, as evident in Figure S2c, is responsible for the higher net silver production rate. The diminished Ag

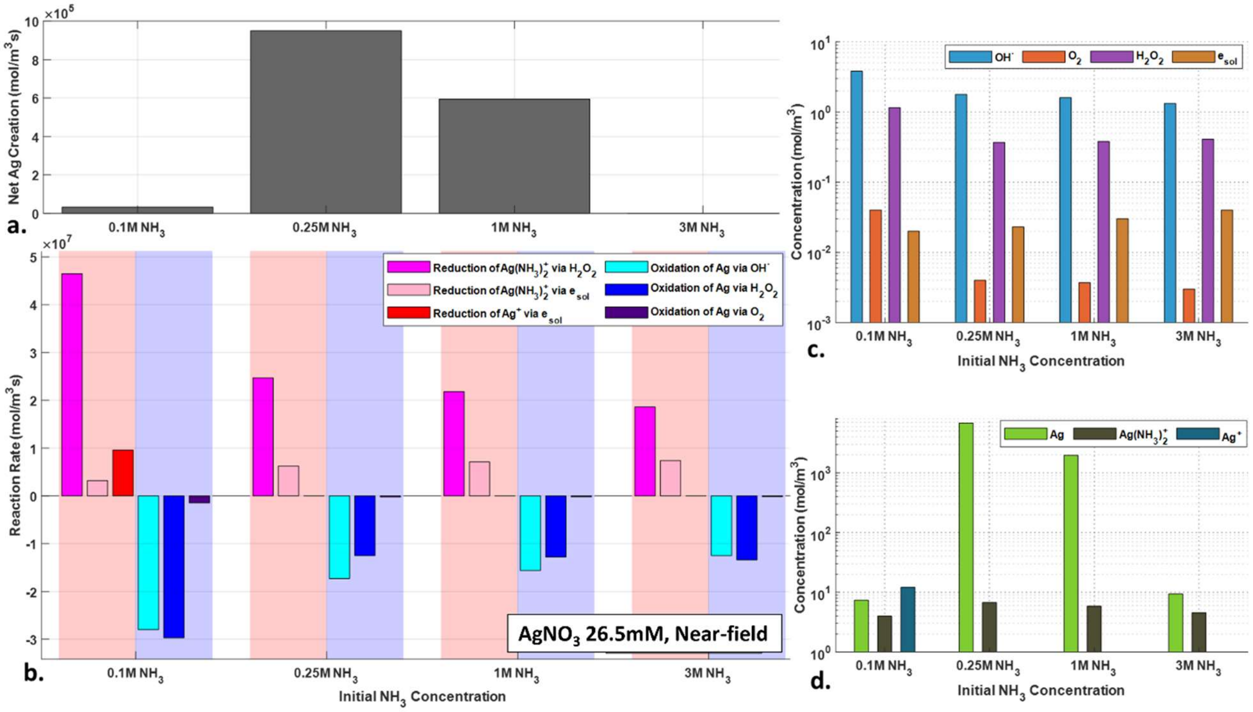

Figure S2. *Key reaction rates and concentrations in the near-field (center) for a 26.5 mM* $AgNO_3$ *solution at* NH_3 *concentrations of 0.1, 0.25, 1, and 3 M post 1 s electron beam irradiation. Panel (a) illustrates the net instantaneous rate of silver deposition showing peak Ag creation in the near-field at 0.25 M* NH_3*. The net Ag silver deposition is the sum of all the reduction and oxidation pathways for precursors and Ag, respectively (b). Concentrations of key oxidizing and reducing species are shown in (c). Concentrations of Ag and the precursor ions are shown in (d), which indicates a partial conversion of* Ag^+ *to* $Ag(NH_3)_2^+$ *contributing to a negligible Ag creation at 0.1 M* NH_3 *(a). Additionally, decreasing precursor concentrations from 0.25 M to 3 M* NH_3 *in (d) is responsible for the drop in Ag creation.*

production with further increases of NH_3 concentrations to 1 M and 3 M corresponds with a decrease in the concentration of $Ag(NH_3)_2^+$ in the near-field, Figure S2d, which is due to consumption of the precursor during far-field silver production which prevents it from being available to diffuse to the center. Figure S3 depicts reaction rates and concentrations of key redox-mediating species in the far-field (4.5 µm from the center) for four representative initial ammonia

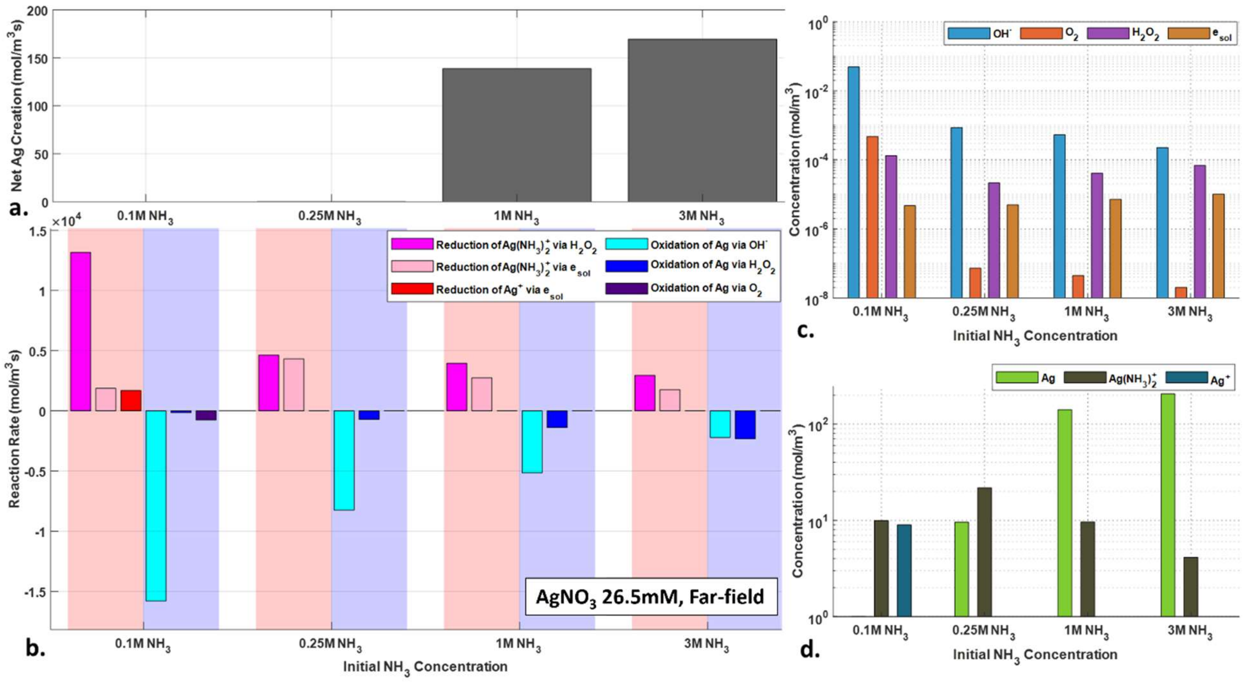

Figure S3. *Key reaction rates and concentrations for far-field (4.5 µm from the center) silver deposition dynamics under varying NH_3 concentrations in a 26.5 mM $AgNO_3$ solution following electron beam exposure of 1 s are shown. The net rate of Ag deposition (a) shows the increase in the rate of Ag creation in the far-field with higher NH_3 concentrations - this rate is the sum of all the reduction and oxidation reaction rates shown in (b). Concentrations of reducing and oxidizing species participating in creation or consumption of Ag is showing in (c). The Ag and precursor ions concentrations (d) show an increase in far-field Ag creation with increased NH_3, as well as a corresponding drop in the precursor ion concentrations.*

concentrations 1 s after electron beam irradiation of a 26.5mM silver nitrate film. As was seen for moderate ammonia concentrations in the near-field, increasing rates of silver production in the far-field at higher ammonia concentrations (Figure S3a) are due primarily not to the increased rates of reduction, but as a result of even greater decrease in the rates of oxidation (Figure S3b). The oxidizing species $OH^{\bullet}$ and O_2 are formed by radiolytic processes in the near-field and must diffuse to the far-field. As NH_3 concentration is raised, more $OH^{\bullet}$ and O_2 are scavenged prior to reaching the far-field, Figure S3c. This leads to a faster drop in the rates of oxidation with increasing ammonia concentration than in the rates of reduction. As a result, net Ag creation occurs in the far-field for these NH_3 concentrations. The reducing environment in the far-field also leads to a drop in concentration of the precursor $Ag(NH_3)_2^+$ as it is consumed in the H_2O_2 mediated reduction reaction, Figure S3d. This leads to a decrease in the rate of $Ag(NH_3)_2^+$ diffusion into the near-field and a slower growth of Ag in the near-field.
